# The Influence of Different Crop Mulches on Weed Infestation, Soil Properties and Productivity of Wheat under Conventional and Conservation Production Systems

**DOI:** 10.3390/plants12010009

**Published:** 2022-12-20

**Authors:** Waqas Ahmed Minhas, Noman Mehboob, Muhammad Yahya, Hafeez Ur Rehman, Shahid Farooq, Mubshar Hussain

**Affiliations:** 1 Department of Agronomy, Bahauddin Zakariya University, Multan 60800, Pakistan; 2 Department of Agronomy, University of Agriculture, Faisalabad 30800, Pakistan; 3 Department of Plant Protection, Faculty of Agriculture, Harran University, Şanlıurfa 63050, Turkey; 4 School of Veterinary and Life Sciences, Murdoch University, 90 South Street, Murdoch, WA 6150, Australia

**Keywords:** wheat, mulches, bulk density, grain yield, economic analysis

## Abstract

Pakistan and other South Asian countries rely on wheat for human nutrition. However, wheat yield is declining in the region due to several biotic and abiotic constraints. Weeds are among the major factors responsible for yield decrease and farmers manage them by intensive tillage practices. Several studies have investigated the impact of various tillage practices on weed management in wheat. However, weed suppression abilities of different mulch types are rarely tested. This three-year (2019–20, 2020–21 and 2021–22) study investigated the impact of different mulch types (prepared from different crops) on weed infestation, soil properties and productivity of wheat under conventional and conservation production systems at three locations (Multan, Hafizabad and Faisalabad) in Punjab, Pakistan. The mulches included in the study were cotton sticks mulch, mungbean straw mulch, sorghum straw mulch, rice straw mulch, sunflower straw mulch, plastic mulch, and no mulch (as control). The production systems opted for wheat cultivation were conventionally tilled wheat (CTW), zero-tilled wheat (ZTW) and zero-tilled wheat sown with happy seeder machine (HSW). The CTW resulted in the lowest soil bulk density and the highest soil porosity after wheat harvest, while ZTW behaved oppositely. Similarly, incorporation of crop mulches resulted in the highest soil porosity and the lowest soil bulk density, while no-mulch incorporation and plastic mulch recorded the highest bulk density and the lowest soil porosity. Regarding mulches by production systems’ interaction, CTW with sorghum straw- and plastic mulches recorded the lowest weed density and biomass, while ZTW with no-mulch recorded the highest weed density and biomass at all locations. The CTW with mungbean straw- and plastic mulches resulted in the highest yield due to significant improvement in yield-related traits. However, ZTW with sorghum straw mulch and no-mulch resulted in the lowest wheat yield. Although sorghum straw mulch suppressed weed infestation, it negatively affected wheat growth. Economic analysis revealed that CTW with mungbean straw mulch resulted in the highest gross and net incomes and benefit:cost ratio (BCR), while the ZTW with rice straw- and sorghum straw mulches produced the lowest gross and net incomes and BCR at all locations. Therefore, mungbean straw mulch is a viable option to improve wheat productivity and net economic returns under different agro-climatic conditions of Punjab, Pakistan.

## 1. Introduction

Wheat (*Triticum aestivum* L.) is an important cereal crop in Pakistan and majority of the country’s population rely on wheat for daily nutrition. Wheat accounts for 1.8% of Pakistan’s gross domestic product (GDP) [1]. Wheat production in the country must be increased by 1.7% annually to meet local demands. However, a 3.9% decrease in the country’s wheat production has been observed recently [2]. Weeds are among the major factors responsible for low wheat productivity [3,4]. Weeds compete with wheat plants and reduce yield by ~34% [5]. Moreover, weeds host several diseases and other pests, which could exert more negative impacts on crop production [6]. Weeds compete with crop plants for light, space, moisture, and nutrients, which reduce yield and quality of the produce [3,5,7]. It has been reported that weed infestation can reduce wheat yield by 25 to 30% depending upon the duration of weed-crop competition, weed species, and weed management practices [8]. Weed infestation is a major hurdle in the adoption of sustainable agriculture [3,4,7].

Conventional tillage (CT) methods are in use for wheat cultivation for several decades. The CT is beneficial in improving soil fertility. However, it deteriorates soil structure, and leads to soil compaction and erosion [9]. Mostly farmers use CT to incorporate previous crops’ residues into the soil [10]. However, it involves several cultivations inverting the entire soil surface, which is not feasible and economical for winter crops [11]. The CT is helpful in weed management during early growth stages of crop plants [12]. However, severe weed infestation is observed at later growth stages. Furthermore, recurrent use of CT at the same depth for seedbed preparation may create plough pan, which reduces crop productivity [13] and results in nutrients’ depletion [14].

Conservation agriculture (CA) practices include minimum tillage (MT) and zero tillage (ZT), which reduce production costs due to lesser tillage and ensure water and soil conservation [15]. The ZT is a viable option for wheat cultivation as it reduces cultivation costs and avoids a 2–3 week delay in crop planting [16]. Reduced or zero tillage is better than CT due to less greenhouse gases’ emissions and better crop yields [17]. However, higher weed infestation is observed in ZT compared to CT [18]. If ZT is practiced without proper soil cover, it can increase infestation of weeds, diseases, and pests, degrade soil structure and lower crop yields [19]. Weeds are a major barrier in the adoption of CA globally [20]. Since tillage is not employed for weed management, cultural practices and integrated weed management are the primary tools for suppressing weed infestation in CA [21].

Mulching is a soil covering technique, which expands soil surface area by adding organic carbon to the soil. Various organic and inorganic materials serve as mulches. The mulches act as a protective layer on the soil surface, conserve soil moisture and suppress weeds’ growth. Mulching helps in maintaining soil moisture and temperature for longer period and suppress weed infestation [22,23]. Different mulches (e.g., rice husk and black polythene sheet) are effective in conserving soil moisture through reducing water use by 3–11% and crop performance by 25%. On an average, 13–21%, 25% and 40% increase in grain yield, root weight and root length have been reported by using mulches [24]. Mulching increases soil temperature and covers the soil, which reduces weed growth [25]. It also reduces weed-crop competition and helps plants in early fruiting and seed setting [26,27]. Moreover, mulching reduces the use of herbicides, improves fertilizer use efficiency [28], reduces soil erosion, and improves grain yield and quality [29]. Mulching combined with deep and shallow cultivation has a great impact on increasing grain yield and water use efficiency [30].

Although various studies have compared the impact of CT and CA on wheat production and soil properties, the impact of different on weed infestation and wheat production is less explored. Moreover, the interactive effect of CT and CA, and different mulch types on soil properties and weed infestation have never been studied in Pakistan. This study investigated the interactive effects of CT and CA with different mulches on wheat productivity, soil properties and weed infestation. It was hypothesized that the mulches of allelopathic crops in CT [(sunflower (*Helianthus annuus* L.) and sorghum (*Sorghum bicolor* L.)] would suppress weed infestation. It was further hypothesized that wheat productivity and soil properties will be improved by the mulches prepared from leguminous crop [mungbean (*Vigna radiata* L.)] in CA. The results would help to improve wheat productivity and economic returns.

## 2. Results

### 2.1. Soil Physical Properties

Soil bulk density and soil porosity were significantly affected by production systems (T), mulches (M) and their interaction T × M at all locations (Table S1). The highest and the lowest soil BD was recorded for zero tilled wheat (ZTW) and conventionally tilled wheat (CTW), respectively. Similarly, CTW observed the highest total soil porosity, whereas ZTW resulted in the lowest values at all locations. Regarding mulches, the highest and the lowest soil BD was noted for no-mulch (N-M) and mungbean straw mulch (mungbean-M), respectively. Likewise, mungbean-M recorded the highest soil porosity, and it was statistically at par with cotton sticks mulch (cotton-M) and sunflower straw mulch (sunflower-M) at Multan and Hafizabad, and rice straw mulch (rice-M) at Faisalabad. The T × M interaction revealed that CTW with mungbean-M recorded the lowest and the highest soil BD and soil porosity, respectively. Likewise, ZTW with N-M recorded the highest soil BD and ZTW with plastic mulch (plastic-M) observed the lowest soil porosity (Table 1).

### 2.2. Total Weed Density and Biomass

The total weed density and biomass were significantly altered by individual and interactive effects of T and M at 45 and 65 days after sowing (DAS) at all locations (Tables S2–S4). The highest and the lowest weed density and biomass were recorded for ZTW and CTW, respectively, at all locations. Wheat sown with plastic-M recorded the lowest weed density and biomass followed by sorghum-M, while N-M observed the highest weed density and biomass at all locations. The T by M interaction indicated that ZTW with N-M observed the highest, while CTW with plastic-M and sorghum-M recorded the lowest density and biomass of weeds (Table 2, Table 3 and Table 4).

### 2.3. Yield-Related Traits of Wheat

Plant height, number of productive tillers, spike length, number of grains per spike and 1000-grain weight were significantly altered by individual effects of T and M at all locations. However, their interactive effect had non-significant on plant height at Multan and Hafizabad, spike length at all locations and number of grains per spike at Multan and Hafizabad (Tables S5–S7). The CTW recorded the highest plant height, number of productive tillers, spike length, number of grains per spike and 1000-grain weight, while ZTW observed the lowest values of these traits. Wheat sown with plastic-M observed the highest plant height, number of productive tillers, spike length, number of grains per spike and 1000-grain weight wheat followed by mungbean-M. However, wheat sown with N-M recorded the lowest values for yield-related traits. Interactive of T × M indicated that CTW with plastic-M recorded the highest values for plant height, number of productive tillers, number of grains per spike and 1000-grain weight followed by mungbean-M at all locations, while ZTW with N-M and sorghum-M recorded the lowest values for yield-related traits (Table 5, Table 6 and Table 7).

Biological, grain and straw yields were significantly altered by the individual and interactive effect of T and M at all locations (Tables S7 and S8). The highest and the lowest values of biological, grain and straw yields were noted for CTW and ZTW, respectively. Furthermore, the highest and the lowest values of biological, grain and straw yields were recorded for Plastic-M and N-M and sorghum-M, respectively. The T × M interaction indicated that CTW with plastic-M and mungbean-M had higher biological, grain and straw yields, whereas ZTW with N-M and sorghum-M recorded the lowest yields (Table 7 and Table 8).

Harvest index was significantly affected by individual and interactive effects of T and M except for non-significant interaction for Multan location (Table S9). The highest and the lowest harvest index was recorded for CTW and ZTW, respectively. Likewise, the highest and the lowest harvest index was recorded for plastic-M and N-M, respectively. The T by M interaction revealed that CTW with mungbean-M and sunflower-M recorded higher, while ZTW with N-M and sorghum-M had lower harvest index (Table 9).

### 2.4. Economic Analysis

Economic analysis indicated that CTW with Plastic-M and mungbean-M recorded the highest gross and net incomes and benefit cost ratio (BCR) at all locations. The CTW with mungbean-M recorded the highest gross and net incomes and BCR at Multan. Similarly, CTW with plastic-M recorded the highest gross and net incomes and BCR at Hafizabad. Likewise, CTW with plastic-M recorded the highest gross and net incomes; however, the highest BCR at Faisalabad was noted for CTW with mungbean-M. The ZTW with N-M resulted in the lowest gross and net incomes at all locations, whereas ZTW with rice-M recorded the lowest BCR at all locations (Table 10).

## 3. Discussion

Different wheat production systems (sowing techniques) and mulches significantly altered the physical properties of soil. The least soil BD and the highest porosity was noted for CTW, while the highest soil BD and the lowest porosity was recorded for ZTW (Table 1). Tillage practices are responsible for the change in soil’s physical characteristics as CTW loosens the soil, while ZTW compacts it. The ZTW conserves soil, minimizes soil BD, penetration resistance, and improves soil organic carbon and soil moisture retention capacity compared to CTW [31]. Heavy tillage practices reduced soil BD and increased soil porosity by 5.19% and 5.69%, respectively. Similarly, CTW had a higher soil porosity and a lower soil BD than no-tillage [32,33]. Reduced tillage intensity improved soil BD, hydraulic conductivity and reduced soil porosity and soil erosion [34]. Reduced tillage practices decrease soil BD, total carbon, and penetration resistances, whereas increased tillage intensity decreases total carbon, improves water infiltration rate and soil porosity [35]. Different crop mulches used in the current study significantly altered soil physical properties. Mungbean-M resulted in the highest soil total porosity and the lowest soil BD, while N-M recorded the highest BD and the lowest porosity. Mulching reduces soil temperature, BD, penetration resistance and improves soil porosity, which promotes root development [36]. The use of mulches improves soil organic carbon, loosens the soil, and alters the soil physical condition (Table 1). Low soil BD and less soil erosion have been observed under mulching compared to no-mulch treatment [37]. Crop residues used as mulch materials add organic matter to the soil. Therefore, crop mulches lowered soil BD along with a significant increase in soil porosity [33]. The Plastic-M changed soil BD, porosity, saturated hydraulic conductivity, and soil moisture contents [38].

Different wheat production systems and mulches significantly altered weed infestation at all locations. The ZTW with N-M recorded the highest weed density and biomass, whereas CTW with plastic-M and sorghum-M recorded the lowest weed density and biomass at all locations (Table 2, Table 3 and Table 4). The intensive tillage in CTW moves the weed seeds to deeper soil layer, which hinders their emergence. Thus, lesser weed infestation is recorded in CTW [39,40]. The deep buried weed seeds in CTW result in lower weed infestation compared to ZT, while ZT system had huge seed bank near to the soil surface, which allows weed emergence and weed-crop competition [41]. The CTW had a great impact on weed flora and proper use of tillage can suppress weed infestation [42]. However, ZT without sufficient soil cover and crop rotation may lead to soil deterioration, infestation of weeds, pests, and diseases, and decreased yields [43]. Many weed seeds are still present on the soil surface in ZT, where they get enough moisture and light to germinate and flourish [44]. The favorable growth conditions [18,19] for the weed seeds bank in the topsoil result in higher weed infestation [45]. Similar findings have been reported by earlier studies [46,47], where ZT observed higher weed infestation, while CT resulted in lower weed infestation.

Mulches are laid on top of the soil to prevent water evaporation and weed growth [48]. Mulches prepared from agricultural residues exert positive impacts on crop productivity because they help in retaining soi moisture, lower soil erosion, and increase organic matter in the soil [49,50]. Different crop mulches included in the current study significantly altered weed infestation in wheat crop. Sorghum-M and Plastic-M significantly suppressed weed flora and resulted in the lowest weed density and biomass at all locations (Table 2, Table 3 and Table 4). Some crops, such as sorghum, rice, and sunflower, had a strong allelopathic effect, which restricts weed growth. Sorghum mulch is known for its strong allelopathic effect [51], which reduces weed density and dry weight by 40.8% and 56.0%. The application of sorghum mulch (5 and 10 kg ha^−1^) reduced weed density by 6 to 43% and biomass by 48 to 66% [52]. Several studies [51,53,54] reported that sunflower and sorghum mulch exhibited strong allelopathic effect and suppressed weed infestation by 67.5 and 67.0%, respectively. These results coincide with our study that sorghum-M significantly suppressed weed infestation in wheat crop. Moreover, Plastic-M blocks sunlight and alters soil temperature, which suppresses weed growth [55], improves crop water use efficiency [56] and suppresses weed infestation [25]. Similarly, densities and dry weights of total weeds were significantly reduced by black plastic mulch [57].

Yield is dependent on early crop performance and if a crop suffers challenges during its early phase, yield and related traits are significantly hampered. The result of the current study revealed that tillage practices and crop mulches significantly affected wheat yield and related traits. The CTW observed the highest, while ZTW observed the lowest values for plant height, number of productive tillers, spike length, number of grains per spike, 1000-grain weight, grain, straw and biological yields, and harvest index at all locations (Table 5, Table 6, Table 7, Table 8 and Table 9). The ZTW recorded the highest soil BD and lowest porosity (Table 1), which restricted root penetration. The weaker root growth in CTW resulted in lesser moisture and nutrient uptake. Thus, resulting in lower yield and related traits. Moreover, ZTW recorded higher weed infestation, which resulted in weed-crop competition for light, space, water and nutrient leading to poor crop performance (Table 2, Table 3 and Table 4). However, the CTW plots had the lowest BD and porosity (Table 1), which help the roots to penetrate deeper for nutrient and moisture extraction leading to better crop growth and yield. Furthermore, CTW had lesser weed infestation and weed-crop competition, which ultimately improved yield and related traits. Lesser weed prevalence results in better yield, while more weed infestation significantly reduced yield [58]. Adoption of suitable sowing techniques can suppress weed infestation, which ultimately affects crop yield [59]. In CTW, soil is inverted [11], which eradicates weeds [12] and makes soil well pulverized, which favors root development and lesser weed-crop competition [60]. Similar findings were reported by Shahzad et al. [61] that CTW observed higher yield as compared to ZTW because it experienced less weed incidence, higher moisture, and nutrient uptake.

Different crop mulches significantly affected wheat yield and related traits. Mungbean-M and Plastic-M recorded the highest values for plant height, number of productive tillers, spike length, number of grains per spike, 1000-grain weight, grain, straw and biological yields, and harvest index at all locations, whereas N-M and sorghum-M recorded the lowest values for these traits (Table 5, Table 6, Table 7, Table 8 and Table 9). Mulches improve soil organic matter content, available P, K and decrease soil pH [8], improve water holding capacity of soil, reduce soil erosion and enhance soil organic matter [47]. Legumes have the tendency to fix atmospheric N_2_ into the soil, which is unavailable to plants [62]. These crops also provide essential nutrients that are readily available to plants [63]. Moreover, mungbean-M also observed lower weed infestation than N-M (Table 2, Table 3 and Table 4). Due to better early crop performance, higher yield and related traits were recorded for mungbean-M. Plastic-M also noted the highest values for yield traits because. The plastic cover did not allow the weeds’ emergence. Hence, the crop produced better yield in the absence of weed-crop competition (Table 2, Table 3 and Table 4). A similar trend has been reported in an earlier study [64] where plastic film mulching increased crop yield by 24%. The ZTW with sorghum-M recorded the lowest yield and related traits due to allelopathic effect of sorghum. Sorghum is highly allelopathic and it not only reduces weed infestation but also exerted negative impacts on wheat yield (Table 2, Table 3, Table 4, Table 5, Table 6, Table 7, Table 8 and Table 9); [51]. Sorghum releases chemicals, including fatty acids, benzoxazinoids, indoles, phenolic acids, phenylalkanoic acids and terpenoids, which had a strong allelochemical effect on suppressing weeds and successive crops [51,54].

It is important how tillage affects nutrient availability after the incorporation of crop residues or their use as surface mulches [65]. Residues’ incorporation may alter nutrient availability because of their inherent nutritional composition [66]. Different crop residues supply varying amounts of nutrients depending on their decomposition speed and nutritional composition [67]. Tillage is another important factor influencing the availability of nutrients, although clay minerology is the most influential parameter in this regard. Several studies have revealed that nutrient supply/release was higher in conventionally tilled soils compared to no tillage [68,69]. The differences in the yield-related attributes in the current study under different production systems can be owed to tillage systems and composition of mulches. The CTW probably released more nutrients compared to ZTW, which improved the crop growth and yield. Similarly, mungbean-M could have higher nutrients than the rest of the crop mulches included in the study, which improved the growth and productivity of wheat crop. However, nutrient release and nutritional composition of the mulches were not tested in the current study. Therefore, these must be examined in future studies to confirm these inferences

Economic analysis revealed that CTW with mungbean-M and plastic-M recorded the highest net and gross incomes, and BCR. The CTW with plastic-M recorded the lowest weed infestation (Table 2, Table 3 and Table 4) and CTW with mungbean-M improved soil physical properties and soil organic carbon both of which produced higher yield. Mungbean-M recorded the highest BCR at all locations as Mungbean-M is cheaper than plastic-M thus, reduced production costs. The ZTW with N-M recorded the lowest gross and net incomes at all locations due to the lowest yield (Table 5, Table 6, Table 7, Table 8 and Table 9).

## 4. Materials and Methods

This three-year study was conducted at a research farm in the Department of Agronomy, Bahauddin Zakariya University Multan, a research farm of the University of Agriculture Faisalabad and at a farmer’s field in district Hafizabad, Pakistan during 2019–20, 2020–21 and 2021–22. The Multan site had loamy soil with 8.31 pH, 2.81 mS cm^−1^ EC, 0.81% organic matter content, 0.103 % total nitrogen (N), 7.85 mg kg^−1^ available phosphorus (P) and 200 mg kg^−1^ available potassium (K). The Hafizabad site had sandy loam soil with pH value of 8.0, 3.20 mS cm^−1^ EC, 0.67% organic matter content, 0.049% total N, 6.78 mg kg^−1^ available P and 175 mg kg^−1^ available K. The Faisalabad site had sandy clay loam soil with pH values of 7.9, EC of 1.35 mS cm^−1^, 0.76% organic matter content, 0.05% total N, 6.74 mg kg^−1^ available P and 181 mg kg^−1^ available K.

### 4.1. Experiment Details

The above ground parts of all kharif crops (cotton, sorghum, mungbean, rice and sunflower) were chaffed properly after harvesting and air dried for 15 days. The crop and plastic mulches were applied after sowing between wheat rows. Crop mulches were applied at the rate 5 t ha^−1^. For CTW, seedbed was prepared by cultivating field two times by tractor mounted cultivator followed by planking to produce well aerated seedbed. For HSM and ZTW, a tractor drawn happy seeder machine and a zero-drill machine were used for wheat sowing without disturbing the soil. This experiment was laid out following RCBD with factorial arrangement and replicated thrice with a net plot size of 5.0 m × 2.7 m.

### 4.2. Crop Husbandry

A pre-soaking irrigation of 10 cm was applied before sowing and seedbed was prepared according to the treatments. The wheat variety ‘Johar-2016′ was sown on November 23 at Multan, November 28 at Faisalabad and December 5 at Hafizabad during 2019–20. Similarly, wheat sowing was done on November 26 at Hafizabad, December 2 at Multan and December 3 at Faisalabad during 2020–21. Likewise, wheat sowing was done on November 23 at Multan, November 25 at Faisalabad and November 30 at Hafizabad during 2021–22. The wheat seed rate was kept at 150 kg ha^−1^ at all locations during all years. Fertilizer was applied at the rate of 150, 100 and 70 kg ha^−1^ of N, P, and K. Nitrogen-based fertilizer was applied in three splits, whereas the whole P and K fertilizers were applied at the time of sowing. Field was irrigated five times during the whole growth period of wheat. For better crop production all cultural and agronomical practices were used to ensure crop safety from pests and diseases. Final crop harvesting was done on 15 April at Multan, 19 April at Hafizabad and 25 April at Faisalabad during the 1st year. During the 2nd year, harvesting was done on 14 April at Multan, 23 April at Hafizabad and 25 April at Faisalabad. During the 3rd year, harvesting was done on 10 April at Multan, 13 April at Hafizabad and 17 April at Faisalabad site. No weed management practices were opted in any of the treatments. The recommended row spacing of 20 cm was used for sowing wheat crop.

### 4.3. Soil Physical Properties

Soil bulk density (BD) and total porosity were analyzed by taking soil samples with soil core sampler after wheat harvest during each year. Three random samples from all experimental plots were taken from 0–15 cm depth, mixed, dried in an oven for 24 h at 105 °C and then BD was measured by following the procedure of Blake and Hartge [70]. Total soil porosity was estimated following Danielson and Sutherland [71].

### 4.4. Data Collection of Weeds

Data regarding totals weed density and biomass were recorded at two different intervals, i.e., 45 and 65 days after sowing of wheat (DAS). Three random places from each experiment unit were selected by using quadrate (1 m × 1 m). All weed plants in the quadrate were uprooted from field and counted (to record density) from each treatment and oven dried for 70 ± 5 °C until the constant weight to record total dry biomass of all weeds.

### 4.5. Agronomic and Yield-Related Traits of Wheat

Data regarding the number of productive tillers were taken from each experimental unit using quadrate method and averaged. The number of spike bearing tillers in each treatment were counted from three different locations in each treatment and averaged. Plant height and spike length (from base to awns) were recorded from 10 randomly selected plants by using measuring tape and averaged. Similarly, the number of grains per spike were counted from 25 randomly selected spikes from each experimental unit. Further, 5 samples of 1000 grains were selected from each experimental unit randomly to record 1000 grain weight. For estimating biological, grain and straw yields, whole experimental units were harvested and sundried for two days and weighed using spring balance to record biological yield. Later, the wheat was threshed manually to obtain grain yield and straw yield using the same weighing balance. Biological, grain and straw yields were converted to t ha^−1^ by unitary method. Harvest index was recorded by using ratio of grain yield to biological yield and expressed in percentage.

### 4.6. Statistical Analysis

The collected data were checked for normality using the Shapiro–Wilk normality test which indicated a normal distribution. Therefore, all statistical analyses were performed on original data. The differences among years were tested by three-way analysis of variance (ANOVA) by taking year as a factor. The ANOVA indicated that the year effect was non-significant. Similarly, the studied locations were far from each other and each in different climatic zones of the country. Therefore, data of different locations were pooled across years and analyzed, presented, and interpreted separately. Two-way ANOVA was used to infer the significance of wheat production systems and mulches for each location separately. The individual effects of wheat production systems and mulches were significant for all recorded traits, while interactions were non-significant for some of the traits. Therefore, both individual and interactive effects were presented and interpreted. Means of individual and interactive effects of mulches and wheat production systems were compared with the least significant difference (LSD) at a 95% probability level where ANOVA indicated significant differences [72]. All statistical computations were made on SPSS statistical software version 21.0.

### 4.7. Economic Analysis

Economic analysis of the current experiment was conducted to estimate the system productivity. The expenditures incurred on wheat production, including land rent, mulches, seed, tillage, labor costs, fertilizers, irrigation, harvesting, etc., were combined to get total expenses. Gross income was computed by using the prevailing market prices of wheat grains and straw. The net income was computed by subtracting the expenditure from gross income. The benefit cost ratio was computed by dividing the net income with the expenses incurred in wheat production. The existing market prices were taken for all the inputs and produce for the relevant years and then averaged across the years. The local currency was then converted into USD keeping in view the current exchange rate.

## 5. Conclusions

The ZTW promoted weed infestation due to least soil disturbance in the upper layer, while CTW suppressed weed infestation. The ZTW with N-M promoted weed infestation, while plastic-M and sorghum-M suppressed weed growth due to soil cover and strong allelopathic effect. The CTW with plastic-M and mungbean-M significantly improved soil physical properties and wheat yield. Moreover, CTW with plastic-M increased wheat yield. However, net benefits of CTW with mungbean-M were higher. Overall, CTW with mungbean-M resulted in the highest economic returns. Therefore, it can be opted for more economic benefits. However, CTW with sorghum-M resulted in the lowest weed infestation; therefore, it can be opted for better weed management in wheat crop.

## Figures and Tables

**Table 1:** Effect of different mulches and production systems on soil bulk density and soil porosity after wheat harvest.

Mulches	CTW	ZTW	HSW	Means (M)	CTW	ZTW	HSW	Means (M)
	2019–20				2020–21			
	Bulk density (g cm^−3^)				Porosity (%)			
	Multan							
Cotton- M	1.42 l	1.51 b	1.47 hi	1.47 C	41.89 bc	37.57 ij	38.62 f–i	39.36 A
Sorghum- M	1.42 kl	1.50 bc	1.48 gh	1.47 C	40.94 cd	35.74 k	38.25 g–j	38.31 C
Mung bean- M	1.38 m	1.48 gh	1.46 j	1.44 D	43.27 a	34.53 lm	39.90 de	39.23 AB
Rice-M	1.43 k	1.51 b	1.49 de	1.48 B	40.75 cd	35.43 kl	38.78 e–h	38.32 C
Sunflower- M	1.42 l	1.49 cd	1.48 ef	1.47 C	42.59 ab	35.07 k–m	39.42 ef	39.03 AB
Plastic- M	1.43 kl	1.50 bc	1.49 de	1.47 B	39.26 e–g	34.22 m	38.11 h–j	37.20 D
N-M	1.47 i	1.53 a	1.48 fg	1.49 A	39.80 de	37.45 j	38.56 f–j	38.60 BC
Means (ST)	1.42 C	1.50 A	1.48 B		41.22 A	35.72 C	38.80 B	
LSD at 5% for ST = 0.02; M = 0.02; ST × M = 0.01					LSD at 5% for ST = 1.15; M = 0.66; ST × M =1.15			
	Hafizabad							
Cotton- M	1.45 k	1.54 bc	1.51 gh	1.50 C	33.05 a–c	29.65 fgh	30.47 d–g	31.06 A
Sorghum- M	1.46 jk	1.53 cd	1.51 fg	1.50 C	32.29 b–d	28.20 hi	30.18 fg	30.23 AB
Mung bean- M	1.41 l	1.51 fg	1.49 i	1.47 D	34.41 a	27.25 i	31.48 c–f	31.05 A
Rice-M	1.46 j	1.54 bc	1.53 de	1.51 B	32.15 b–e	27.95 hi	30.59 d–g	30.23 AB
Sunflower- M	1.45 k	1.54 b	1.52 e	1.51 B	33.61 ab	27.67 i	31.09 d–g	30.79 A
Plastic- M	1.45 k	1.56 a	1.53 de	1.51 B	30.98 d–g	27.00 i	30.06 fg	29.35 B
N-M	1.49 h	1.56 a	1.52 ef	1.52 A	31.39 c–f	29.54 gh	30.42 e–g	30.45 A
Means (ST)	1.45 C	1.54 A	1.51 B		32.56 A	28.18 C	30.61 B	
LSD at 5% for ST = 0.02; M = 0.01; ST × M = 0.01					LSD at 5% for ST = 0.69; M = 1.06; ST × M = 1.83			
	Faisalabad							
Cotton- M	1.44 lm	1.54 a–c	1.50 g–i	1.49 C	36.54 bc	32.39 h–k	33.95 f–h	34.29 BC
Sorghum- M	1.45 kl	1.53 b–d	1.51 f–i	1.49 C	36.45 bc	29.83 m	34.06 e–h	33.44 CD
Mung bean- M	1.41 n	1.51 f–i	1.49 j	1.47 E	39.16 a	32.11 i–l	35.86 c–e	35.71 A
Rice-M	1.46 k	1.54 ab	1.52 b–f	1.51 B	36.28 b–d	31.54 j–m	34.53 d–g	34.11 B–D
Sunflower- M	1.45 kl	1.53 b–e	1.52 d–g	1.49 BC	37.92 ab	31.22 k–m	35.09 c–g	34.74 AB
Plastic- M	1.43 m	1.51 e–h	1.49 h–j	1.48 D	34.95 c–g	30.47 lm	33.93 f–i	33.11 D
N-M	1.49 ij	1.56 a	1.52 c–f	1.53 A	35.43 c–f	33.34 g–j	33.95 f–h	34.24 BC
Means (ST)	1.45 C	1.53 A	1.51 B		36.67 A	31.55 C	34.48 B	
LSD at 5% for ST= 0.02; M = 0.01; ST×M = 0.02					LSD at 5% for ST = 0.69; M = 1.06; ST×M = 1.84			

Means of various parameters followed by different case letters (uppercase for individual effects and lowercase for interactions) within columns or rows significantly differ from each other at *p* ≤ 0.05. Here, M = mulches, ST = sowing techniques, CTW = conventionally tilled wheat, ZTW = zero tilled wheat, and HSW= Happy seeder drilled wheat.

**Table 2:** Effect of different mulches and sowing techniques on total weed density and biomass at Multan.

Mulches	CTW	ZTW	HSW	Means (M)	CTW	ZTW	HSW	Means (M)
	Multan							
	Total weed density (plants m^−2^)				Total weed biomass (g m^−2^)			
	45 DAS							
Cotton- M	10.67 fg	25.33 d	17.67 e	17.89 C	1.81 g–i	4.53 d	3.37 e	3.24 B
Sorghum- M	3.33 ij	11.00 fg	7.00 h	7.11 E	0.29 k	1.56 g–j	0.92 h–k	0.92 D
Mungbean- M	7.67 gh	20.67 e	12.00 f	13.44 D	1.74 g–i	3.04 ef	1.84 gh	2.21 C
Rice-M	12.33 f	28.67 d	19.33 e	20.11 B	2.38 fg	5.00 cd	3.35 e	3.58 B
Sunflower- M	5.00 hi	14.00 f	8.00 gh	9.00 E	0.63 jk	1.72 g–i	0.86 i–k	1.07 D
Plastic- M	0.67 j	5.33 hi	2.67 ij	2.89 F	0.04 k	0.67 jk	0.26 k	0.32 E
N-M	33.33 c	73.00 a	49.33 b	51.89 A	5.56 c	11.7 a	8.06 b	8.45 A
Means (ST)	10.43 C	25.43 A	16.57 B		1.78 C	4.04 A	2.67 B	
LSD at 5% for ST = 1.34; M = 2.05; ST × M = 3.56					LSD at 5% ST= 0.36; M = 0.56; ST × M = 0.97			
	65 DAS							
Cotton- M	29.00 i	58.33 e	44.33 g	43.88 C	11.85 f	22.52 d	17.79 e	17.39 B
Sorghum- M	12.67 kl	31.33 i	21.67 j	21.88 F	4.09 kl	9.92 gh	7.04 ij	7.02 E
Mungbean- M	23.33 j	54.00 f	36.67 h	38.00 D	7.78 i	17.61 e	12.45 f	12.61 C
Rice-M	31.67 i	65.33 d	45.00 g	47.33 B	12.26 f	23.48 d	16.27 e	17.34 B
Sunflower- M	15.33 k	36.67 h	25.00 j	25.67 E	5.20 jk	11.33 fg	8.09 hi	8.21 D
Plastic- M	4.33 m	16.00 k	9.67 l	10.00 G	1.19 m	4.96 k	3.03 lm	3.06 F
N-M	72.00 c	126.67 a	89.33 b	96.00 A	26.64 c	45.50 a	32.60 b	34.91 A
Means (ST)	26.90 C	55.48 A	38.81 B		9.86 C	19.33 A	13.89 B	
LSD at 5% for ST = 1.30; M = 1.99; ST × M = 3.44					LSD at 5% ST = 0.72; M = 1.11; ST × M = 1.92			

Means of various parameters followed by different case letters (uppercase for individual effects and lowercase for interactions) within columns or rows significantly differ from each other at *p* ≤ 0.05. Here, M = mulches, ST = sowing techniques, CTW = conventionally tilled wheat, ZTW = zero tilled wheat, and HSW= Happy seeder drilled wheat.

**Table 3:** Effect of different mulches and sowing techniques on total weed density and biomass at Hafizabad.

Mulches	CTW	ZTW	HSW	Means (M)	CTW	ZTW	HSW	Means (M)
	Hafizabad							
	Total weed density (plants m^−2^)				Total weed biomass (g m^−2^)			
	45 DAS							
Cotton- M	12.00 h	30.67 d	20.33 f	21.00 C	2.17 hi	6.36 de	4.06 f	4.19 C
Sorghum- M	4.00 jk	15.67 g	8.67 i	9.44 E	0.66 j–l	3.01 gh	1.51 ij	1.73 E
Mungbean- M	11.67 h	29.00 de	19.67 f	20.11 C	2.19 hi	5.92 e	3.80 fg	3.97 C
Rice-M	15.33 g	38.67 c	26.33 e	26.78 B	2.96 gh	7.97 c	5.46 e	5.46 B
Sunflower- M	7.33 i	19.33 f	12.00 h	12.89 D	1.39 i–k	3.71 fg	2.41 hi	2.50 D
Plastic- M	1.00 l	6.67 ij	3.33 kl	3.67 F	0.05 l	1.04 j–l	0.43 kl	0.51 F
N-M	37.67 c	79.00 a	58.67 b	58.44 A	7.31 cd	18.07 a	12.21 b	12.53 A
Means (ST)	12.71 C	31.29 A	21.29 B		2.39 C	6.58 A	4.27 B	
LSD at 5% for ST = 1.07; M = 1.63; ST × M = 2.83					LSD at 5% ST = 0.59; M = 0.39; ST × M = 1.03			
	65 DAS							
Cotton- M	29.00 i	57.00 d	40.00 fg	42.00 C	11.89 j	23.74 e	16.45 gh	17.36 C
Sorghum- M	15.00 k	36.00 gh	23.33 j	24.78 F	6.08 l	15.43 hi	9.79 k	10.43 F
Mungbean- M	26.33 ij	50.00 e	36.33 gh	37.56 D	11.47 j	22.15 e	15.51 h	16.38 D
Rice-M	34.33 h	61.00 cd	46.67 e	47.33 B	13.89 i	25.73 d	19.52 f	19.72 B
Sunflower- M	18.67 k	41.33 f	28.00 i	29.33 E	7.66 l	17.51 g	11.66 j	12.28 E
Plastic- M	5.33 l	15.00 k	9.33 l	9.89 G	2.18 n	6.24 l	4.09 m	4.17 G
N-M	64.00 c	123.33 a	91.33 b	92.89 A	27.58 c	53.79 a	40.33 b	40.57 A
Means (ST)	27.52 C	54.81 A	39.29 B		11.54 C	23.51 A	16.76 B	
LSD at 5% for ST = 1.53; M = 2.35; ST × M = 4.06					LSD at 5% ST = 0.60; M = 0.92; ST × M = 1.60			

Means of various parameters followed by different case letters (uppercase for individual effects and lowercase for interactions) within columns or rows significantly differ from each other at *p* ≤ 0.05. Here, M = mulches, ST = sowing techniques, CTW = conventionally tilled wheat, ZTW = zero tilled wheat, and HSW= Happy seeder drilled wheat.

**Table 4:** Effect of different mulches and sowing techniques on total weed density and biomass at Faisalabad.

Mulches	CTW	ZTW	HSW	Means (M)	CTW	ZTW	HSW	Means (M)
	Faisalabad							
	Total weed density				Total weed biomass			
	45 DAS							
Cotton- M	11.67 g–i	29.00 c	19.33 de	20.00 B	2.64 gh	6.72 d	4.64 e	4.67 B
Sorghum- M	5.67 k–m	16.33 ef	10.67 h–j	10.89 D	1.19 jk	3.60 f	2.32 hi	2.37 D
Mungbean- M	8.67 i–k	22.00 d	14.67 fg	15.11 C	1.89 ij	4.99 e	3.17 fg	3.35 C
Rice-M	11.67 g–i	26.67 c	19.00 de	19.11 B	3.13 fg	6.37 d	4.88 e	4.79 B
Sunflower- M	7.67 j–l	21.33 d	13.33 f–h	14.11 C	1.51 jk	4.81 e	3.24 fg	3.19 C
Plastic- M	2.00 m	7.00 j–l	4.00 lm	4.33 E	0.25 l	1.44 jk	0.83 kl	0.84 E
N-M	30.00 c	60.33 a	40.00 b	43.44 A	7.65 c	15.49 a	11.13 b	11.42 A
Means (ST)	11.05 C	26.09 A	17.29 B		2.61 C	6.20 A	4.32 B	
LSD at 5% for ST = 1.45; M = 2.21; ST × M = 3.83					LSD at 5% ST =0.27; M = 0.41; ST × M = 0.72			
	65 DAS							
Cotton- M	23.33 i	47.67 c	33.00 fg	34.67 B	9.79 i	20.73 c	14.17 fg	14.89 B
Sorghum- M	13.00 m	31.00 g	20.00 j	21.33 F	4.95 lm	13.37 g	8.28 j	8.87 F
Mungbean- M	18.67 jk	39.67 e	26.33 h	28.22 D	7.87 jk	17.53 e	11.31 h	12.24 D
Rice-M	23.00 i	44.33 d	31.33 g	32.89 C	9.69 i	18.99 d	13.45 g	14.04 C
Sunflower- M	16.33 kl	35.33 f	23.67 i	25.11 E	6.88 k	15.21 f	10.22 hi	10.77 E
Plastic- M	5.67 o	14.33 lm	10.00 n	10.00 G	1.92 n	5.44 l	3.88 m	3.75 G
N-M	42.00 de	77.00 a	58.00 b	59.00 A	18.13 de	33.59 a	24.89 b	25.54 A
Means (ST)	20.29 C	41.33 A	28.90 B		8.46 C	17.84 A	12.32 B	
LSD at 5% for ST = 1.44; M = 0.94; ST × M = 2.50					LSD at 5% ST = 0.42; M = 0.65; ST × M = 1.12			

Means of various parameters followed by different case letters (uppercase for individual effects and lowercase for interactions) within columns or rows significantly differ from each other at *p* ≤ 0.05. Here, M = mulches, ST = sowing techniques, CTW = conventionally tilled wheat, ZTW = zero tilled wheat, and HSW= Happy seeder drilled wheat.

**Table 5:** Effect of different mulches and sowing techniques on plant height and number of productive tillers of wheat.

Mulches	CTW	ZTW	HSW	Means (M)	CTW	ZTW	HSW	Means (M)
	Plant Height (cm)				Number of Productive Tillers (m^−2^)			
	Multan							
Cotton- M	107.00 ^NS^	100.77	104.07	103.94 B	172.50 de	155.13 f–h	157.57 e–g	161.73 CD
Sorghum- M	108.10	98.70	100.00	102.27 C	169.33 d–f	157.73 e–g	141.00 h–j	156.02 D
Mung bean- M	114.60	103.87	107.43	108.63 A	223.00 b	163.13 ef	179.93 d	188.69 B
Rice-M	108.90	99.73	104.30	104.31 B	180.53 d	138.23 ij	166.53 d–f	161.77 CD
Sunflower- M	108.03	101.13	103.40	104.19 B	205.70 c	142.77 g–i	159.13 ef	169.20 C
Plastic- M	115.20	103.13	106.43	108.26 A	243.90 a	167.33 d–f	199.10 c	203.44 A
N-M	102.33	94.50	99.33	98.72 D	139.87 h–j	126.70 j	131.53 ij	132.70 E
Means (ST)	109.17 A	100.26 C	103.57 B		190.69 A	150.15 C	162.11 B	
LSD at 5% for ST = 1.06; M = 1.63; ST × M = NS					LSD at 5% for ST = 6.02; M = 9.19; ST × M =15.92			
	Hafizabad							
Cotton- M	103.63 d	94.73 ij	99.50 g	99.29 D	212.40 c	158.67 ij	176.73 fg	182.60 D
Sorghum- M	100.50 e–g	90.70 k	94.90 ij	95.37 E	192.67 de	141.50 k	164.40 hi	166.19 E
Mungbean- M	108.00 b	100.97 e–f	102.97 d	103.98 B	229.17 b	178.17 f	189.33 de	198.89 B
Rice-M	105.50 c	97.40 h	100.20 e–g	101.03 C	227.67 b	164.17 hi	180.87 f	190.90 C
Sunflower- M	102.67 d	95.67 i	99.83 fg	99.39 D	214.50 c	160.87 i	170.33 gh	181.90 D
Plastic-M	109.73 a	101.37 e	105.00 c	105.37 A	252.33 a	195.37 d	212.67 c	220.12 A
N-M	99.37 g	89.87 k	94.07 j	94.43 F	188.00 e	132.00 l	152.33 j	157.44 F
Means (ST)	104.20 A	95.81 C	99.50 B		216.68 A	161.53 C	178.10 B	
LSD at 5% for ST = 0.46; M = 0.69; ST × M = 1.21					LSD at 5% for ST = 2.47; M = 3.77; ST × M = 6.53			
	Faisalabad							
Cotton- M	103.47 ^NS^	97.33	100.50	100.43 D	214.00 de	178.97 hi	197.83 fg	196.93 C
Sorghum- M	101.80	94.10	97.27	97.72 E	195.17 g	164.50 jk	162.50 k	174.06 D
Mungbean- M	106.53	99.13	101.93	102.53 B	243.33 b	189.17 h	210.00 d–f	214.17 B
Rice-M	106.43	97.20	101.23	101.62 C	221.17 cd	177.00 h–j	202.83 e–g	200.33 C
Sunflower- M	102.90	97.13	99.90	99.98 D	232.10 bc	167.9 i jk	193.93 g	197.99 C
Plastic- M	109.77	102.63	105.03	105.81 A	284.00 a	217.17 d	241.33 b	247.50 A
N-M	100.60	92.63	96.80	96.68 F	198.33 fg	154.17 k	166.17 i–k	172.89 D
Means (ST)	104.50 A	97.17 C	100.38 B		226.87 A	178.41 C	196.37 B	
LSD at 5% for ST = 0.57; M = 0.88; ST × M = NS					LSD at 5% for ST = 5.37; M = 8.19; ST × M = 14.20			

Means of various parameters followed by different case letters (uppercase for individual effects and lowercase for interactions) within columns or rows significantly differ from each other at *p* ≤ 0.05. Here, M = mulches, ST = sowing techniques, CTW = conventionally tilled wheat, ZTW = zero tilled wheat, and HSW= Happy seeder drilled wheat.

**Table 6:** Effect of different mulches and sowing techniques on spike length and number of grains per plant of wheat.

Mulches	CTW	ZTW	HSW	Means (M)	CTW	ZTW	HSW	Means (M)
	Spike Length (cm)				Number of Grains Per Spike			
	Multan							
Cotton- M	10.67 ^NS^	9.47	9.97	10.03 B	48.47 ^NS^	43.37	45.77	45.87 C
Sorghum- M	9.70	8.97	9.33	9.33 C	45.27	41.53	44.73	43.84 D
Mung bean- M	11.40	9.83	10.53	10.59 A	50.93	46.10	47.73	48.26 B
Rice-M	10.77	9.40	10.13	10.10 B	49.10	43.13	46.80	46.34 C
Sunflower- M	10.40	9.23	9.93	9.86 B	48.60	43.60	46.67	46.29 C
Plastic- M	11.50	9.96	10.77	10.74 A	53.20	46.80	49.93	49.98 A
N-M	9.60	8.70	9.33	9.21 C	44.57	40.57	43.53	42.89 E
Means (ST)	10.58 A	9.37 C	10.00 B		48.59 A	43.59 C	46.45 B	
LSD at 5% for ST = 0.18; M = 0.28; ST × M = NS					LSD at 5% for ST = 0.51; M = 0.78; ST × M = NS			
	Hafizabad							
Cotton- M	10.83 ^NS^	9.93	10.70	10.48 D	48.27 ^NS^	42.53	45.03	45.28 D
Sorghum- M	10.80	9.43	10.40	10.21 E	45.83	41.33	41.93	43.03 E
Mung bean- M	11.70	10.27	10.97	10.98 B	49.97	44.80	47.40	47.39 B
Rice-M	11.43	10.10	10.70	10.74 C	49.03	43.03	46.80	46.29 C
Sunflower- M	11.13	9.73	10.07	10.31 DE	47.53	42.33	44.03	44.63 D
Plastic- M	12.40	11.07	11.50	11.65 A	54.33	47.93	50.30	50.86 A
N-M	10.53	9.13	9.67	9.78 F	44.60	39.70	42.10	42.13 F
Means (ST)	11.26 A	9.95 C	10.57 B		48.51 A	43.09 C	45.37 B	
LSD at 5% for ST = 0.14; M = 0.21; ST × M = NS					LSD at 5% for ST = 0.48; M = 0.74; ST × M = NS			
	Faisalabad							
Cotton- M	11.00 ^NS^	9.90	10.47	10.46 C	50.10 cd	44.13 jk	47.60 efg	47.28 C
Sorghum- M	10.53	9.43	9.97	9.98 D	47.43 fg	41.37 l	44.17 jk	44.32 D
Mung bean- M	11.77	10.30	10.90	10.99 B	53.17 b	47.10 gh	49.00 de	49.76 B
Rice-M	11.83	10.23	10.93	11.00 B	50.97 c	44.50 ijk	48.70 def	48.06 C
Sunflower- M	11.07	9.53	10.37	10.32 C	50.77 c	44.90 ij	47.03 gh	47.57 C
Plastic- M	12.33	10.83	11.60	11.58 A	56.26 a	48.53 ef	51.40 c	52.07 A
N-M	10.47	9.40	9.57	9.81 D	45.83 hi	44.63 ij	43.13 k	44.53 D
Means (ST)	11.29 A	9.95 C	10.54 B		50.65 A	45.02 C	47.29 B	
LSD at 5% for ST = 0.13; M = 0.19; ST × M = NS					LSD at 5% for ST = 0.54; M = 0.83; ST × M = 1.43			

Means of various parameters followed by different case letters (uppercase for individual effects and lowercase for interactions) within columns or rows significantly differ from each other at *p* ≤ 0.05. Here, M = mulches, ST = sowing techniques, CTW = conventionally tilled wheat, ZTW = zero tilled wheat, and HSW= Happy seeder drilled wheat.

**Table 7:** Effect of different mulches and sowing techniques on 1000-grain weight (g) and biological yield (t ha^−1^) of wheat.

Mulches	CTW	ZTW	HSW	Means (M)	CTW	ZTW	HSW	Means (M)
	Thousand Grain Weight (g)				Biological Yield (t ha^−1^)			
	Multan							
Cotton- M	41.60 c–e	39.47 h–j	40.30 f–h	40.46 BC	16.40 bc	12.84 kl	13.98 gh	14.41 D
Sorghum- M	40.30 f–h	37.90 l	40.17 f–i	39.46 D	14.02 gh	11.25 n	12.39 lm	12.56 E
Mung bean- M	45.13 a	40.93 ef	42.47 bc	42.84 A	18.55 a	14.69 f	15.76 de	16.34 B
Rice-M	43.00 b	38.93 jk	40.63 e–g	40.86 B	16.63 bc	12.99 jk	13.43 ij	14.35 D
Sunflower- M	42.07 b–d	38.97 jk	39.70 g–j	40.24 C	16.88 b	13.29 ijk	14.46 fg	14.88 C
Plastic- M	44.87 a	41.10 d–f	42.00 cd	42.66 A	19.06 a	15.51 e	16.27 cd	16.95 A
N-M	39.80 g–j	38.23 kl	39.23 ij	39.09 D	13.66 hi	10.96 n	12.04 m	12.22 F
Means (ST)	42.39 A	39.36 C	40.64 B		16.46 A	13.08 C	14.05 B	
LSD at 5% for ST = 0.36; M = 0.56; ST × M = 0.98					LSD at 5% for ST = 0.20; M = 0.31; ST × M =0.54			
	Hafizabad							
Cotton- M	40.33 de	37.07 k	37.97 ij	38.46 D	12.80 c	9.60 k	10.83 hi	11.08 D
Sorghum- M	39.23 fg	35.70 m	38.17 hi	37.70 E	11.03 gh	8.63 m	9.57 kl	9.74 F
Mung bean- M	42.03 b	38.80 gh	40.50 d	40.44 B	13.90 b	10.67 i	11.80 de	12.12 B
Rice-M	41.37 c	37.50 jk	38.93 g	39.27 C	13.57 b	10.13 j	11.60 ef	11.77 C
Sunflower- M	40.67 d	37.17 k	38.03 ij	38.62 D	12.13 d	9.23 l	10.67 i	10.68 E
Plastic- M	42.97 a	39.70 ef	40.87 cd	41.18 A	15.40 a	11.30 fg	12.57 c	13.09 A
N-M	38.70 gh	36.40 l	36.90 kl	37.33 E	11.10 gh	8.30 m	9.43 kl	9.61 F
Means (ST)	40.76 A	37.48 C	38.77 B		12.85 A	9.69 C	10.92 B	
LSD at 5% for ST = 0.24; M = 0.37; ST × M = 0.64					LSD at 5% for ST = 0.13; M = 0.20; ST × M = 0.35			
	Faisalabad							
Cotton- M	40.13 cd	36.87 k	37.73 ij	38.24 D	12.67 c	9.87 i	10.97 gh	11.17 C
Sorghum- M	39.00 ef	35.47 l	37.93 hi	37.47 E	11.47 ef	8.77 k	9.97 i	10.07 D
Mung bean- M	41.63 b	38.57 fgh	40.27 c	40.15 B	14.40 b	10.67 h	11.77 de	12.28 B
Rice-M	41.40 b	37.27 jk	38.73 fg	39.13 C	14.33 b	10.87 gh	12.03 d	12.41 B
Sunflower- M	40.47 c	36.93 k	38.27 ghi	38.56 D	13.03 c	9.87 i	11.13 fg	11.34 C
Plastic- M	42.73 a	39.47 de	40.63 c	40.94 A	16.267 a	11.40 ef	12.80 c	13.49 A
N-M	38.47 f–h	35.80 l	36.87 k	37.04 F	11.37 f	8.40 k	9.37 j	9.71 E
Means (ST)	40.55 A	37.19 C	38.633 B		13.36 A	9.97 C	11.15 B	
LSD at 5% for ST = 0.26; M = 0.39; ST × M = 0.68					LSD at 5% for ST = 0.14; M = 0.22; ST × M = 0.38			

Means of various parameters followed by different case letters (uppercase for individual effects and lowercase for interactions) within columns or rows significantly differ from each other at *p* ≤ 0.05. Here, M = mulches, ST = sowing techniques, CTW = conventionally tilled wheat, ZTW = zero tilled wheat, and HSW= Happy seeder drilled wheat.

**Table 8:** Effect of different mulches and sowing techniques on grain and straw yields of wheat.

Mulches	CTW	ZTW	HSW	Means (M)	CTW	ZTW	HSW	Means (M)
	Grain Yield (t ha^−1^)				Straw Yield (t ha^−1^)			
	Multan							
Cotton- M	5.07 b	4.17 f	4.47 e	4.57 C	11.35 cd	8.68 jk	9.50 f–h	9.84 D
Sorghum- M	4.43 e	3.87 gh	3.97 g	4.09 D	9.62 fg	7.38 m	8.43 kl	8.48 E
Mung bean- M	5.50 a	4.43 e	4.87 c	4.93 B	13.06 a	10.24 e	10.91 d	11.40 B
Rice-M	5.07 b	4.23 f	4.50 de	4.60 C	11.59 bc	8.78 ijk	8.90 i–k	9.76 D
Sunflower- M	5.03 b	4.23 f	4.47 e	4.58 C	11.85 b	9.06 hij	9.98 ef	10.30 C
Plastic- M	5.60 a	4.60 d	5.00 b	5.07 A	13.48 a	10.94 d	11.26 cd	11.89 A
N-M	4.47 e	3.77 h	3.93 g	4.06 D	9.22 ghi	7.17 m	8.13 l	8.17 F
Means (ST)	5.02 A	4.18 C	4.46 B		11.45 A	8.89 C	9.59 B	
LSD at 5% for ST = 0.04; M = 0.06; ST × M = 0.11					LSD at 5% for ST = 0.18; M = 0.28; ST × M = 0.49			
	Hafizabad							
Cotton- M	4.27 d	3.17 h	3.50 g	3.64 E	8.57 c	6.40 i	7.30 fg	7.42 C
Sorghum- M	3.73 f	2.73 j	3.17 h	3.21 F	7.30 fg	5.90 jk	6.40 i	6.53 E
Mung bean- M	4.83 b	3.63 fg	4.00 e	4.16 B	9.13 b	7.03 gh	7.77 de	7.98 B
Rice-M	4.67 c	3.30 h	3.77 f	3.91 C	8.90 bc	6.83 h	7.83 de	7.86 B
Sunflower- M	4.57 c	3.20 h	3.57 g	3.78 D	7.57 ef	6.03 j	7.10 gh	6.90 D
Plastic- M	5.67 a	4.10 e	4.57 c	4.78 A	9.80 a	7.17 gh	8.00 d	8.32 A
N-M	3.57 g	2.60 j	3.00 i	3.06 G	7.53 ef	5.67 k	6.43 i	6.54 E
Means (ST)	4.47 A	3.25 C	3.65 B		8.40 A	6.43 C	7.26 B	
LSD at 5% for ST = 0.05; M = 0.08; ST × M = 0.15					LSD at 5% for ST = 0.13; M = 0.20; ST × M = 0.36			
	Faisalabad							
Cotton- M	4.43 c	2.97 k	3.67 h	3.69 D	8.20 c	6.87 ef	7.27 de	7.44 C
Sorghum- M	4.03 de	2.80 l	3.37 ij	3.40 E	7.47 d	5.97 hi	6.60 fg	6.68 D
Mung bean- M	4.87 b	3.37 ij	4.13 d	4.12 B	9.50 b	7.23 de	7.63 d	8.12 B
Rice-M	4.87 b	3.47 i	3.93 ef	4.09 B	9.43 b	7.43 d	8.07 c	8.31 B
Sunflower- M	4.73 b	3.27 j	3.70 gh	3.90 C	8.30 c	6.60 fg	7.40 d	7.43 C
Plastic- M	5.30 a	3.80 f–h	4.37 c	4.49 A	10.93 a	7.63 d	8.43 c	9.00 A
N-M	3.83 fg	2.63 m	3.03 k	3.17 F	7.50 d	5.73 i	6.37 gh	6.53 D
Means (ST)	4.58 A	3.18 C	3.74 B		8.76 A	6.78 C	7.39 B	
LSD at 5% for ST= 0.06; M = 0.09; ST × M = 0.16					LSD at 5% for ST = 0.15; M = 0.23; ST × M = 0.40			

Means of various parameters followed by different case letters (uppercase for individual effects and lowercase for interactions) within columns or rows significantly differ from each other at *p* ≤ 0.05. Here, M = mulches, ST = sowing techniques, CTW = conventionally tilled wheat, ZTW = zero tilled wheat, and HSW= Happy seeder drilled wheat.

**Table 9:** Effect of different mulches and sowing techniques on harvest index of wheat.

Mulches	CTW	ZTW	HSW	Means (M)
	Harvest Index			
	Multan			
Cotton- M	35.16 ^NS^	38.00	35.50	36.22 A
Sorghum- M	32.10	35.53	29.57	32.40 BC
Mung bean- M	36.86	33.06	33.10	34.34 AB
Rice-M	32.80	22.30	33.47	29.52 C
Sunflower- M	35.00	32.90	32.77	33.56 AB
Plastic- M	35.60	33.77	34.03	34.47 AB
N-M	32.43	29.77	30.23	30.81 BC
Means (ST)	34.28	32.19	32.67	
LSD at 5% for ST = 2.45; M = 3.75; ST × M = NS				
	Hafizabad			
Cotton- M	31.57 de	27.97 hi	30.97 d–g	30.17 D
Sorghum- M	29.43 gh	25.60 jk	29.83 fg	28.29 E
Mung bean- M	34.33 b	29.93 e–g	30.87 d–g	31.71 C
Rice-M	32.43 cd	31.17 d–f	29.80 fg	31.13 CD
Sunflower- M	36.20 a	29.83 fg	32.33 d	32.79 B
Plastic- M	34.70 ab	34.10 bc	34.33 b	34.38 A
N-M	30.47 efg	24.43 k	26.97 ij	27.29 F
Means (ST)	32.73 A	29.00 C	30.73 B	
LSD at 5% for ST = 0.65; M = 0.99; ST × M = 1.72				
	Faisalabad			
Cotton- M	35.13 ab	26.53 k	31.37 e–h	31.01 CD
Sorghum- M	32.30 d–f	27.43 k	31.20 f–i	30.31 D
Mung bean- M	36.20 a	30.46 g–j	33.90 bc	33.52 A
Rice-M	34.00 bc	29.60 j	30.31 g–j	31.31 C
Sunflower- M	35.37 ab	29.77 j	31.50 e–h	32.21 B
Plastic- M	33.40 cd	31.80 efg	32.70 c–e	32.63 B
N-M	31.23 e–i	30.70 g–j	30.17 h–j	30.70 CD
Means (ST)	33.95 A	29.47 C	31.59 B	
LSD at 5% for ST = 0.56; M = 0.86; ST × M = 1.48				

Means of various parameters followed by different case letters (uppercase for individual effects and lowercase for interactions) within columns or rows significantly differ from each other at *p* ≤ 0.05. Here, M = mulches, ST = sowing techniques, CTW = conventionally tilled wheat, ZTW = zero tilled wheat, and HSW= Happy seeder drilled wheat.

**Table 10:** Economic analysis of wheat under different surface mulches and sowing techniques.

Surface Mulch	CTW	ZTW	HSW	CTW	ZTW	HSW	CTW	ZTW	HSW	CTW	ZTW	HSW
	Cost of Production (US$)			Gross Income (US$)			Net Income (US$)			BCR		
	Multan											
Cotton- M	730	668	668	1780	1512	1634	1050	844	966	2.44	2.26	2.45
Sorghum- M	724	662	662	1600	1427	1518	875	764	856	2.21	2.15	2.29
Mung bean- M	718	657	657	1929	1664	1766	1211	1008	1109	2.69	2.53	2.69
Rice-M	893	831	831	1808	1534	1642	915	703	811	2.03	1.85	1.98
Sunflower- M	730	668	668	1870	1503	1648	1141	835	980	2.56	2.25	2.47
Plastic- M	807	745	745	1918	1647	1776	1112	902	1031	2.38	2.21	2.38
N-M	657	595	595	1664	1320	1450	1007	725	856	2.53	2.22	2.44
	Hafizabad											
Cotton- M	730	668	668	1532	1146	1277	802	478	609	2.10	1.72	1.91
Sorghum- M	724	662	662	1336	1005	1140	612	343	478	1.85	1.52	1.72
Mung bean- M	718	657	657	1706	1291	1427	987	635	770	2.37	1.97	2.17
Rice-M	893	831	831	1658	1194	1359	765	364	529	1.86	1.44	1.64
Sunflower- M	730	668	668	1576	1130	1281	846	462	613	2.16	1.69	1.92
Plastic- M	807	745	745	1962	1428	1588	1155	683	843	2.43	1.92	2.13
N-M	657	595	595	1303	963	1095	646	368	501	1.98	1.62	1.84
	Faisalabad											
Cotton- M	730	668	668	1568	1110	1323	839	442	656	2.15	1.66	1.98
Sorghum- M	724	662	662	1419	1021	1202	694	359	540	1.96	1.54	1.82
Mung bean- M	718	657	657	1743	1240	1452	1024	584	796	2.43	1.89	2.21
Rice-M	893	831	831	1734	1266	1422	841	435	591	1.94	1.52	1.71
Sunflower- M	730	668	668	1651	1179	1336	921	511	668	2.26	1.76	2.00
Plastic- M	807	745	745	1925	1363	1554	1118	618	809	2.39	1.83	2.09
N-M	657	595	595	1378	970	1101	721	375	506	2.10	1.63	1.85

## Data Availability

All data are within the manuscript.

## Supplementary Materials

The following supporting information can be downloaded at: https://www.mdpi.com/article/10.3390/plants12010009/s1, Table S1: Analysis of variance for the individual and interactive effect of different mulches and production systems on soil bulk density and soil porosity after wheat harvest; Table S2: Analysis of variance for the individual and interactive effect of different mulches and production systems on weed density and biomass in wheat crop at Multan; Table S3: Analysis of variance for the individual and interactive effect of different mulches and production systems on weed density and biomass in wheat crop at Hafizabad; Table S4: Analysis of variance for the individual and interactive effect of different mulches and production systems on weed density and biomass in wheat crop at Faisalabad; Table S5: Analysis of variance for the individual and interactive effect of different mulches and production systems on plant height and number of productive tillers of wheat; Table S6: Analysis of variance for the individual and interactive effect of different mulches and production systems on spike length and number of grains per spike of wheat; Table S7: Analysis of variance for the individual and interactive effect of different mulches and production systems on thousand grain weight and biological yield of wheat; Table S8: Analysis of variance for the individual and interactive effect of different mulches and production systems on grain and straw yields of wheat; Table S9: Analysis of variance for the individual and interactive effect of different mulches and production systems on harvest index of wheat.
