# Supplementary material for: The Influence of Different Crop Mulches on Weed Infestation, Soil Properties and Productivity of Wheat under Conventional and Conservation Production Systems
Source: Plants (Basel). 2022 Dec 20;12(1):9. doi: 10.3390/plants12010009 (PMC9824819; doi:10.3390/plants12010009)
Supplement: Supplementary file 1 [file plants-12-00009-s001.zip › plants-2065236-supplementary.pdf]

plants

Supplementary Materials

Table S1. Analysis of variance for the individual and interactive effect of different mulches and production systems on soil bulk density and soil porosity after wheat harvest.

Location	SOV	DF	Sum of Squares	Mean Squares	F value	*P* value
			Soil bulk density (g cm^{-3})			
Faisalabad	Mulches (M)	6	0.01831	0.00305	27.57	0.003**
	Production systems (P)	2	0.07493	0.03747	338.59	0.0012**
	M × P	12	0.00317	0.00026	2.39	0.0197*
Hafizabad	Mulches (M)	6	0.01582	0.00264	80.08	0.0000**
	Production systems (P)	2	0.08213	0.04106	1247.39	0.0000**
	M × P	12	0.00393	0.00033	9.94	0.0000**
Multan	Mulches (M)	6	0.01512	0.00252	94.66	0.0200*
	Production systems (P)	2	0.07183	0.03591	1349.32	0.0010**
	M × P	12	0.00425	0.00035	13.30	0.0300*
			Soil porosity (%)			
Faisalabad	Mulches (M)	6	38.934	6.489	5.22	0.0205*
	Production systems (P)	2	277.033	138.516	111.47	0.0310*
	M × P	12	33.771	2.814	2.2	0.0265*
Hafizabad	Mulches (M)	6	19.373	3.229	2.61	0.0313*
	Production systems (P)	2	201.880	100.940	81.64	0.0000**
	M × P	12	31.541	2.628	2.13	0.0371*
Multan	Mulches (M)	6	29.600	4.933	10.14	0.0000**
	Production systems (P)	2	319.296	159.648	328.14	0.03000*
	M × P	12	47.544	3.962	8.14	0.02000*

SOV = sources of variation, DF = degree of freedom, ** = the relevant individual and interactive effect is significant ($p < 0.01$), * = the relevant individual and interactive effect is significant ($p < 0.05$)

Table S2. Analysis of variance for the individual and interactive effect of different mulches and production systems on weed density and biomass in wheat crop at Multan.

SOV	DF	Sum of Squares	Mean Squares	F value	*P* value
Weed density at 45 DAS					
Mulches (M)	6	411.284	68.547	199.57	0.0200*
Production systems (P)	2	54.262	27.131	78.99	0.0250*
M × P	12	33.169	2.764	8.05	0.0400*
Weed density at 65 DAS					
Mulches (M)	6	14397.0	2399.51	516.55	0.0230*
Production systems (P)	2	2388.3	1194.14	257.07	0.0400*
M × P	12	1237.0	103.09	22.19	0.0320*
Weed biomass at 45 DAS					
Mulches (M)	6	5966.88	994.48	737.13	0.0010**
Production systems (P)	2	948.46	274.229	351.51	0.0200*
M × P	12	247.87	20.656	15.31	0.0310*
Weed biomass at 65 DAS					
Mulches (M)	6	41771.3	6961.88	1597.23	0.0400*
Production systems (P)	2	8650.8	4325.40	992.35	0.0300*
M × P	12	1879.0	156.58	35.92	0.0250*

SOV = sources of variation, DF = degree of freedom, * = the relevant individual and interactive effect is significant ($p < 0.05$)

Table S3. Analysis of variance for the individual and interactive effect of different mulches and production systems on weed density and biomass in wheat crop at Hafizabad.

SOV	DF	Sum of Squares	Mean Squares	F value	*P* value
Weed density at 45 DAS					
Mulches (M)	6	839.76	139.96	361.63	0.0200*
Production systems (P)	2	185.33	92.664	239.42	0.0400*
M × P	12	92.02	7.669	19.81	0.0230*
Weed density at 65 DAS					
Mulches (M)	6	17387.7	2897.94	983.15	0.0300*
Production systems (P)	2	3628.6	1814.29	615.51	0.0400*
M × P	12	1203.2	100.27	34.02	0.0330*
Weed biomass at 45 DAS					
Mulches (M)	6	7136.05	1189.34	1265.64	0.0300*
Production systems (P)	2	1514.95	757.47	806.06	0.0200*
M × P	12	420.96	35.08	37.33	0.0300*
Weed biomass at 65 DAS					
Mulches (M)	6	37000.1	6166.68	1016.62	0.0100*
Production systems (P)	2	7866.9	3933.44	648.45	0.0220*
M × P	12	2125.3	177.11	29.20	0.0420*

SOV = sources of variation, DF = degree of freedom, * = the relevant individual and interactive effect is significant ($p < 0.05$)

Table S4. Analysis of variance for the individual and interactive effect of different mulches and production systems on weed density and biomass in wheat crop at Faisalabad.

SOV	DF	Sum of Squares	Mean Squares	F value	*P* value
Weed density at 45 DAS					
Mulches (M)	6	620.385	103.397	545.35	0.0200*
Production systems (P)	2	135.974	67.987	358.58	0.0330*
M × P	12	39.414	3.285	17.32	0.0400*
Weed density at 65 DAS					
Mulches (M)	6	8219.9	1369.99	253.59	0.0300*
Production systems (P)	2	2400.7	1200.33	222.19	0.0400*
M × P	12	582.4	48.54	8.98	0.0330*
Weed biomass at 45 DAS					
Mulches (M)	6	2429.33	404.888	879.30	0.0300*
Production systems (P)	2	932.71	466.354	1012.79	0.0200*
M × P	12	116.52	9.71	21.09	0.0230*
Weed biomass at 65 DAS					
Mulches (M)	6	12357.7	2059.62	899.52	0.0230*
Production systems (P)	2	4702.3	2351.16	1026.85	0.0420*
M × P	12	569.0	47.42	20.71	0.0260*

SOV = sources of variation, DF = degree of freedom, * = the relevant individual and interactive effect is significant ($p < 0.05$)

Table S5. Analysis of variance for the individual and interactive effect of_different mulches and production systems on plant height and number of productive tillers of wheat.

Location	SOV	DF	Sum of Squares	Mean Squares	F value	*P* value
Plant height (cm)						
Faisalabad	Mulches (M)	6	503.74	83.957	99.01	0.0300*
	Production systems (P)	2	567.53	283.766	334.64	0.0200*
	M × P	12	15.33	1.278	1.51	0.1621^{NS}
Hafizabad	Mulches (M)	6	889.55	148.259	277.28	0.0200*
	Production systems (P)	2	742.03	371.015	693.9	0.0420*
	M × P	12	18.34	1.529	2.86	0.0203*
Multan	Mulches (M)	6	628.22	104.703	35.91	0.0010**
	Production systems (P)	2	851.03	425.517	145.95	0.0200*
	M × P	12	69.59	5.799	1.99	0.0518*
Number of productive tillers m^{-2}						
Faisalabad	Mulches (M)	6	34888.5	5814.7	78.52	0.0400*
	Production systems (P)	2	25205.6	12602.8	170.18	0.0200*
	M × P	12	2334.5	194.5	2.63	0.0190*
Hafizabad	Mulches (M)	6	23296.4	3882.7	247.78	0.0170*
	Production systems (P)	2	33624.6	16812.3	1072.88	0.0300*
	M × P	12	472.7	39.4	2.51	0.0244*
Multan	Mulches (M)	6	28373.5	4728.91	50.78	0.0200*
	Production systems (P)	2	18224.7	9112.33	97.85	0.0000**
	M × P	12	7572.1	631.01	6.78	0.0300*

SOV = sources of variation, DF = degree of freedom, ** = the relevant individual and interactive effect is significant ($p < 0.01$), * = the relevant individual and interactive effect is significant ($p < 0.05$), NS = non-significant

Table S6. Analysis of variance for the individual and interactive effect of different mulches and production systems on spike length and number of grains per spike of wheat.

Location	SOV	DF	Sum of Squares	Mean Squares	F value	*P* value
			Spike length (cm)			
Faisalabad	Mulches (M)	6	21.5660	3.59434	81.66	0.0200*
	Production systems (P)	2	18.8765	9.43825	214.43	0.0020*
	M × P	12	0.7635	0.06362	1.45	0.1863NS
Hafizabad	Mulches (M)	6	19.8063	3.30106	67.76	0.03000*
	Production systems (P)	2	18.0238	9.01190	185.00	0.02000*
	M × P	12	1.0917	0.09098	1.87	0.06940 NS
Multan	Mulches (M)	6	17.9749	2.99582	34.28	0.0000**
	Production systems (P)	2	15.3724	7.68619	87.94	0.0000**
	M × P	12	0.9965	0.08304	0.95	0.5096 NS
			Number of grains per spike			
Faisalabad	Mulches (M)	6	405.341	67.557	89.68	0.0000**
	Production systems (P)	2	336.248	168.124	223.18	0.0200*
	M × P	12	50.901	4.242	5.63	0.0300*
Hafizabad	Mulches (M)	6	458.237	76.373	127.14	0.0200*
	Production systems (P)	2	310.402	155.201	258.37	0.0320*
	M × P	12	11.240	0.937	1.56	0.1437NS
Multan	Mulches (M)	6	316.334	52.722	78.40	0.0000**
	Production systems (P)	2	264.858	132.429	196.93	0.0300*
	M × P	12	14.729	1.227	1.83	0.0769NS

SOV = sources of variation, DF = degree of freedom, ** = the relevant individual and interactive effect is significant ($p < 0.01$), * = the relevant individual and interactive effect is significant ($p < 0.05$), NS = non-significant

Table S7. Analysis of variance for the individual and interactive effect of different mulches and production systems on thousand grain weight and biological yield of wheat.

Location	SOV	DF	Sum of Squares	Mean Squares	F value	*P* value
			1000-grain weight (g)			
Faisalabad	Mulches (M)	6	105.975	17.6625	104.75	0.0300*
	Production systems (P)	2	118.797	59.3987	352.28	0.0220*
	M × P	12	4.954	0.4128	2.45	0.0169*
Hafizabad	Mulches (M)	6	106.264	17.7107	116.24	0.0300*
	Production systems (P)	2	114.744	57.3719	376.56	0.0100**
	M × P	12	5.825	0.4854	3.19	0.0290*
Multan	Mulches (M)	6	115.098	19.1830	54.63	0.0300*
	Production systems (P)	2	97.390	48.6948	138.68	0.0000**
	M × P	12	15.333	1.2777	3.64	0.0230*
			Biological yield (t ha^{-1})			
Faisalabad	Mulches (M)	6	97.024	16.1707	302.39	0.0300*
	Production systems (P)	2	124.169	62.0843	1160.97	0.0200*
	M × P	12	6.143	0.5119	9.57	0.0220*
Hafizabad	Mulches (M)	6	86.909	14.4848	318.07	0.0200*
	Production systems (P)	2	106.036	53.0178	1164.21	0.0000**
	M × P	12	3.291	0.2743	6.02	0.0340*
Multan	Mulches (M)	6	166.492	27.7487	256.69	0.0000**
	Production systems (P)	2	127.465	63.7325	589.57	0.0000**
	M × P	12	3.828	0.3190	2.95	0.0350*

SOV = sources of variation, DF = degree of freedom, ** = the relevant individual and interactive effect is significant ($p < 0.01$), * = the relevant individual and interactive effect is significant ($p < 0.05$)

Table S8. Analysis of variance for the individual and interactive effect of different mulches and production systems on grain and straw yields of wheat.

Location	SOV	DF	Sum of Squares	Mean Squares	F value	*P* value
			Grain yield (t ha^{-1})			
Faisalabad	Mulches (M)	6	11.1238	1.8540	205.09	0.0210*
	Production systems (P)	2	20.7165	10.3583	1145.86	0.0110*
	M × P	12	0.3124	0.0260	2.88	0.0260*
Hafizabad	Mulches (M)	6	18.1787	3.02979	370.99	0.0000*
	Production systems (P)	2	16.3267	8.16333	999.59	0.0200*
	M × P	12	0.5556	0.0463	5.67	0.0330*
Multan	Mulches (M)	6	7.8689	1.31148	280.08	0.0100*
	Production systems (P)	2	7.6803	3.84016	820.10	0.0200*
	M × P	12	0.2797	0.02331	4.98	0.0231*
			Straw yield (t ha^{-1})			
Faisalabad	Mulches (M)	6	42.8743	7.1457	119.63	0.0230*
	Production systems (P)	2	43.1851	21.5925	361.5	0.0290*
	M × P	12	5.0038	0.4170	6.98	0.0200*
Hafizabad	Mulches (M)	6	28.0521	4.6753	100.67	0.0300*
	Production systems (P)	2	40.9470	20.4735	440.82	0.0230*
	M × P	12	2.2641	0.1887	4.06	0.0190*
Multan	Mulches (M)	6	102.582	17.0970	186.45	0.0220*
	Production systems (P)	2	73.649	36.8245	401.59	0.0300*
	M × P	12	3.268	0.2723	2.97	0.0048*

SOV = sources of variation, DF = degree of freedom, ** = the relevant individual and interactive effect is significant ($p < 0.01$), * = the relevant individual and interactive effect is significant ($p < 0.05$)

Table S9. Analysis of variance for the individual and interactive effect of different mulches and production systems on harvest index of wheat.

Location	SOV	DF	Sum of Squares	Mean Squares	F value	*P* value
			Harvest index			
Faisalabad	Mulches (M)	6	72.017	12.003	14.81	0.0180*
	Production systems (P)	2	210.564	105.282	129.93	0.0200*
	M × P	12	78.201	6.517	8.04	0.0300*
Hafizabad	Mulches (M)	6	330.558	55.093	50.70	0.0320*
	Production systems (P)	2	146.250	73.1249	67.30	0.0400*
	M × P	12	68.855	5.7379	5.28	0.0380*
Multan	Mulches (M)	6	286.93	47.822	3.08	0.0143*
	Production systems (P)	2	50.42	25.2097	1.62	0.2103^{NS}
	M × P	12	309.27	25.7721	1.66	0.1142^{NS}

SOV = sources of variation, DF = degree of freedom, * = the relevant individual and interactive effect is significant ($p < 0.05$), NS = non-significant
